# Comprehensive Analysis of HERV Transcriptome in HIV+ Cells: Absence of HML2 Activation and General Downregulation of Individual HERV Loci

**DOI:** 10.3390/v12040481

**Published:** 2020-04-23

**Authors:** Nicole Grandi, Maria Paola Pisano, Sante Scognamiglio, Eleonora Pessiu, Enzo Tramontano

**Affiliations:** 1 Department of Life and Environmental Sciences, University of Cagliari, Cittadella Universitaria di Monserrato-SS554, 09042 Monserrato, Cagliari, Italy; mp.pisano@unica.it (M.P.P.); s.scognamiglio@studenti.unica.it (S.S.); e.pessiu@studenti.unica.it (E.P.); tramon@unica.it (E.T.); 2 Istituto di Ricerca Genetica e Biomedica, Consiglio Nazionale delle Ricerche (CNR), 09042 Monserrato, Cagliari, Italy

**Keywords:** HERV, HIV, endogenous retroviruses, RNAseq, HERV expression, HML2

## Abstract

Human endogenous retrovirus (HERV) expression is currently studied for its possible activation by HIV infection. In this context, the HERV-K(HML2) group is the most investigated: it has been proposed that HIV-1 infection can prompt HML2 transcription, and that HML2 proteins can affect HIV-1 replication, either complementing HIV or possibly influencing antiretroviral therapy. However, little information is available on the expression of other HERV groups in HIV infection. In the present study, we used a bioinformatics pipeline to investigate the transcriptional modulation of approximately 3250 well-characterized HERV loci, comparing their expression in a public RNA-seq profile, including a HIV-1-infected and a control T cell culture. In our pilot study, we found approximately 200 HERV loci belonging to 35 HERV groups that were expressed in one or both conditions, with transcripts per million (TPM) values from 1 to >500. Intriguingly, HML2 elements constituted only the 3% of expressed HERV loci, and in most cases (160) HERV expression was downregulated in the HIV-infected culture, showing from a 1- to 14-fold decrease as compared to uninfected cells. HERV transcriptome has been inferred de novo and employed to predict a total of about 950 HERV open reading frames (ORFs). These have been validated according to the coding potential and estimated abundance of the corresponding transcripts, leading to a set of 57 putative proteins potentially encoded by 23 HERV loci. Analysis showed that some individual loci have a coding potential that deserves further investigation. Among them, a HML6 provirus at locus 19q13.43 was predicted to produce a transcript showing the highest TPM among HERV-derived transcripts, being upregulated in HIV+ cells and inferred to produce Gag and Env puteins with possible biological activity.

## 1. Introduction

Human endogenous retroviruses (HERVs) are genomic remnants of past infections, sustained by ancestral exogenous retroviruses that became extinct millions of years ago. Such ancient viruses have been able to integrate their proviral genome within primate germ line DNA, and have been hence inherited through the offspring, forming a remarkable proportion of our genetic material (~8%). HERVs belong to the ensemble of human transposable elements, constituting the Long Terminal Repeat (LTR) provided retrotransposons. During evolution, some HERV loci have been domesticated and provide important biological functions to the host physiology [1]. These include promoter activity for cellular genes, evolution of pivotal immune pathways and protein cooptation for placenta development and functions [2,3]. In addition, HERV expression is currently of great interest for the possible pathological significance of their RNA and proteins [1]. In fact, it has been suggested that HERVs could have a role in a number of complex human disorders, such as cancer and autoimmunity, even if, to date, no specific HERV loci have been clearly linked to any of these conditions [1]. Another main field of HERV investigation is related to their possible interplay with exogenous infections, which could hypothetically lead to either beneficial or harmful effects to the host. Among the favorable effects, it has been hypothesized that HERV transcripts might trigger cellular antiviral defenses through the formation of double strand RNAs (dsRNA) between antisense homologous mRNA sequences, activating innate immunity effectors [4,5]. Another putative antiviral effect exerted by HERV proteins might be based on receptor interference and blocking, which confer partial resistance to exogenous infections, as observed with some animal ERVs [6,7]. On the contrary, harmful interactions between exogenous viruses and HERV products might include reciprocal upregulation of viral transcription or complementation of defective proteins [8]. Of course, most of these interactions imply a certain degree of sequence and protein homology and are more prone to occur between endogenous and exogenous retroviruses. 

While HERV relics reveal the presence of more than 30 ancestral main retroviral groups belonging to the beta-, gamma-, and epsilon genera [9], modern humans are threatened by only two exogenous retroviruses: human immunodeficiency virus (HIV, genus Lentivirus) and human T cell lymphotropic virus (HTLV, genus Deltaretrovirus). However, due to the greater relevance for human epidemiology, the existing literature on the possible interplay with HERVs is mostly limited to HIV infection. In the latter, most of the studies address the possible interaction between HIV and the HERV-K(HML2) group, which is the most recently acquired group with many members still retaining intact open reading frames (ORFs) and protein coding potential [10], while little information is available for the interplay with other HERV groups. In general, it has been suggested that HIV infection can modulate HERV loci transcription, possibly leading to the increase of HERV transcripts and, eventually, stimulating the production of HERV proteins that could either complement [11,12] or interfere [13,14,15] with HIV replication. Such scenarios lead also to the hypothesis that HERV expression can influence antiviral immune responses or have an effect on antiretroviral therapy in HIV patients [16,17,18,19,20,21]. 

Focusing on the possible effects of HIV infection on HERV transcription, current results are still discordant: on the one side there is a body of literature reporting that HIV infection increases the expression of endogenous elements [22,23,24,25,26,27] while, on the other side, a number of studies propose the absence of such stimulation by HIV [28,29]. It is worth noting that all these studies assess the overall, general expression of one or more genes of a given HERV group, i.e., without connecting the observed transcript to any specific locus and without information regarding primer specificity and cross-reactivity. Hence, it is currently difficult to extrapolate if the observed HERV expression in the presence or absence of HIV infection is sustained by the same HERV loci, and it is difficult to clearly establish a common effect of HIV-mediated modulation, if any. The few studies analyzing the effect of HIV on the expression of individual HERV loci focus on a subset of HML2 elements, showing either the absence of modulation [30] or the presence of some specific loci that are stimulated by HIV presence [30,31], also depending on the biological compartment taken into account [32]. Overall, such a specific, multifaceted modulation suggests a complex regulation that cannot be ascertained without considering the individual HERV loci, asking for procedures able to take into account the whole HERV-transcriptome and to distinguish the expression of highly similar sequences, such as next generation sequencing. In fact, one of the main obstacles in characterizing the HERV loci specific transcription is their nucleotide identity, which prevents the univocal discrimination by approaches such as PCR or microarray, often leading to variable conclusions [30].

In the present study, we rely on a comprehensive HERV dataset characterizing in detail the genomic position and structural integrity of ~3250 individual HERV loci, which have been classified in 31 canonical HERV groups plus additional 39 HERV clades showing high degrees of mosaicism [9,33,34,35,36,37] (as described in Supplementary Material, File S1). Taking advantage of such an exhaustive genomic background, we are able to investigate the specific expression of each HERV locus in a publicly available dataset including raw RNA sequencing (RNA-seq) profiles obtained from a human T-cell line (H9) culture seven days after HIV infection and a control uninfected culture (GEO accession number: GSE70785) [38]. This RNA-seq dataset was previously used to assess pseudogene expression in the presence of HIV infection [38] and, even if lacking biological replicates, had technical characteristics that made it optimal for the pilot analysis of HERV transcriptional activity (see Materials and Methods for further details). The characterization of individual HERV loci expression reveal that a proportion of elements are transcriptionally active in human T cells, being mostly downregulated in the presence of HIV infection. The same modulation has been observed for the inferred transcripts, which show a residual coding capacity and hence a possible biological significance. Overall, our study, even if performed on a limited number of samples, provides an exhaustive description of HERV expression in the presence of HIV, identifying specific candidates worthy of further investigation in a large sample set.

## 2. Materials and Methods 

### 2.1. RNA-Sequencing Dataset

Raw RNA-seq data analyzed in this study were publicly available at the NCBI’s Gene Expression Omnibus (GEO) database, accession number GSE70785 [38]. The dataset includes the RNA-seq profiles from two human T-cell line H9 cultures, a HIV-infected one (SRR2096825) and a non-infected control (SRR2096826). The cellular RNA has been sequenced 7 days post-infection, generating paired-end reads of 150 + 150 bp (approximately 80 million reads/sample) [38]. All the details regarding culturing, sequencing and downstream quality control of the dataset can be found elsewhere [38]. Raw RNA-seq data have been downloaded in fastq format and subjected to further quality checks before analyses.

### 2.2. HERV Reference Database

The above RNA-seq dataset has been analyzed for HERV expression using as a background an exhaustive database that includes a total of 3251 univocal HERV sequences, each of which has been characterized in terms of genomic position, group of belonging, nucleotide sequence and predicted coding capacity. Such a HERV reference dataset derives from the recent classification and characterization of the most integer HERV insertions within the human genome, as performed with the software RetroTector [9], further integrated with the comprehensive characterization of specific HERV groups of relevance based on the current literature [33,34,35], with particular attention to HML2 elements (89 loci, [10]) (File S1). 

### 2.3. Bioinformatic Pipeline for HERV Expression Analysis

Raw RNA-seq data have been mapped to the human reference genome sequence (GRCh38/hg38) using STAR aligner, version 2.5.2 [39]. Then, the Python library htseq-count [40] has been used to quantify the reads mapping to each individual HERV locus included in the above dataset, relying on its univocal genomic coordinates. The same framework has been used to count the reads mapping to all the human genes included in Gencode dataset, version 29 [41]. Raw counts have been analyzed with RStudio software, version 1.2.1335 [42] and the relative abundance of reads has been calculated as transcripts per million Kb (TPM) expression values for HERV and cellular genes analyzed. TPM values have been compared statistically in terms of fold changes because a differential expression analysis requires at least 3 samples for each condition. A detailed description of the pipeline is available as Supplementary Material, File S1.

### 2.4. De Novo Trasnscript Reconstruction and Protein Prediction

Raw RNA-seq data have been used for the de novo reconstruction of HIV-infected and uninfected cell culture transcriptomes using Trinity software [43,44]. The obtained transcript abundance has been quantified with Salmon [45] and expressed as TPM values as well. Reconstructed transcripts have been mapped back to the human genome reference sequence with GMAP mRNA aligner [46] to evaluate their structure and compare the expression levels of HERV-derived transcripts between the two conditions. Finally, TransDecoder utility [44] has been used to identify candidate coding regions within transcript sequences and to predict the resulting products. The obtained peptides have been matched through BLASTp [47] with a collection of putative consensus proteins (puteins) as obtained previously for the various HERV groups through the software RetroTector [9,48], to identify HERV-related products. These have been visualized in the context of the putatively encoding HERV loci and transcripts within the Integrative Genomics Viewer (IGV) [49]. A detailed description of the pipeline is available as Supplementary Material, File S1.

## 3. Results

### 3.1. Landscape of HERV Expression in HIV-Infected and Uninfected T Cells

**Figure 1:**
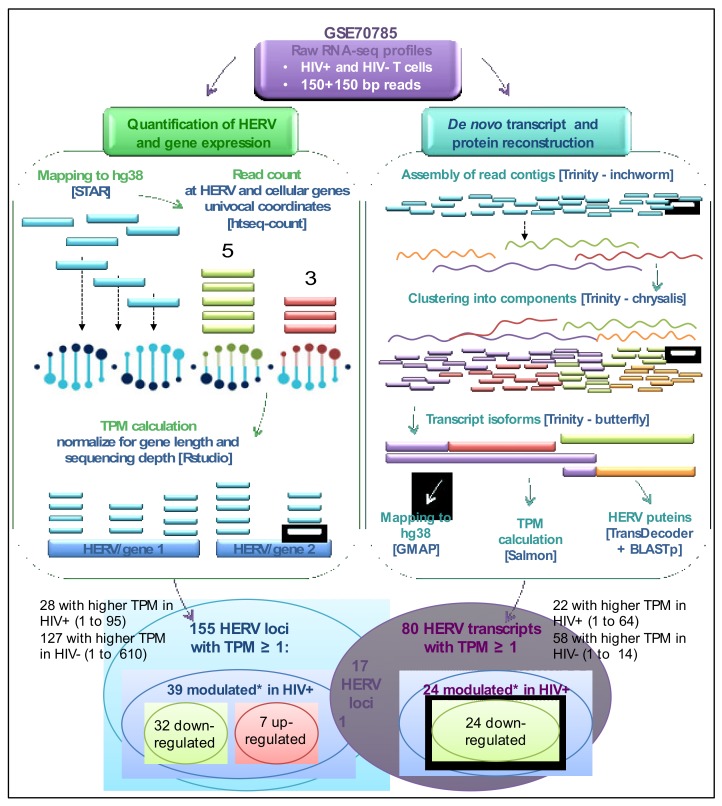
Graphical overview of the bioinformatics pipeline and the identified expressed human endogenous retrovirus (HERV) loci and transcripts. Modulation is based on at least a 3-fold change in the transcripts per million (TPM) value between the two conditions.

**Figure 2:**
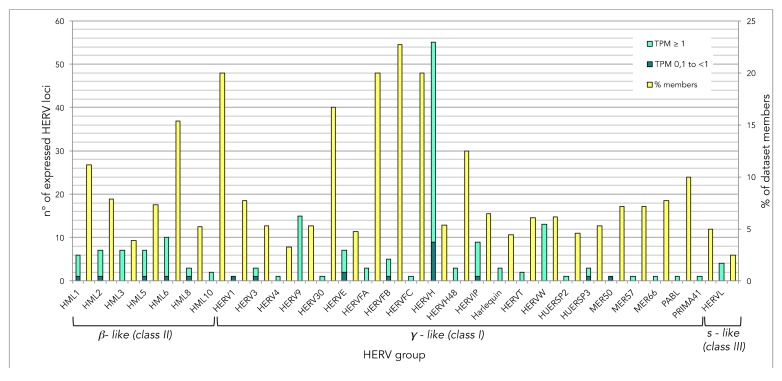
Landscape of HERV loci expressed (TPM value ≥ 0.1) in one or both conditions (HIV- and/or HIV+ T cells), divided in taxonomic viral groups. The number of expressed members (left axis) as well as their percentage with respect to the group total members (right axis) are reported.

The specific expression of approximately 3250 individual HERV loci, as classified and exhaustively characterized in previous studies [9,10,33,34,35], has been analyzed in a public dataset including raw RNA-seq profiles from a HIV-infected and an uninfected human H9 T-cell culture [50], using a classical bioinformatics pipeline for expression analyses optimized for HERV detection. A description of such a pipeline can be found in Materials and Methods as well as in Supplementary Material (File S1), while a schematic representation is provided in Figure 1. Results showed that a total of 177 HERV loci (~5% of the whole dataset) were expressed in at least one condition, with transcripts per million (TPM) expression values from 0.1 to 610 (mean TPM = 8.8; median TPM= 1.85; Supplementary Material, File S2). In general, the expressed HERV loci showed higher TPM values in the uninfected cell culture (mean TPM = 11.3; median TPM = 2.2), being mostly downregulated (146 out of 177) in the presence of HIV infection (mean TPM = 6.3; median TPM = 1.5; File S2). The expressed HERV loci belong to a total of 30 HERV groups, including members from Class I (γ-like, 131 HERV loci from 22 groups), Class II (β-like, 45 HERV loci from 7 groups) and Class III (spuma-like, 4 HERV-L loci) (Figure 2). Overall, percentages of expressed loci in relation to the total number of group members ranged from 2.5% (HERV-L) to 23% (HERV-FB) (Figure 2). Around 77% of expressed HERV loci were colocalized with known cellular genes based on Gencode annotations (File S2). 

To corroborate subsequent analyses, we set a cutoff of minimum expression and took into account those HERV loci with TPM value ≥1 in at least one condition. We included in this way a total of 155 HERV loci from the above 177 expressed sequences. In general, these 155 HERV loci showed higher TPM values in uninfected cells (mean TPM = 12.8; median TPM = 2.7), being mostly downregulated in the presence of HIV infection (144 out of 155 loci: mean TPM 7.2; median TPM = 1.8; Figure 1 and File S2). In more detail, 28 HERV loci showed the highest expression in HIV+ cells (mean TPM = 7.3 vs 5.5; median TPM = 1.6 vs 0.9; fold change range = 1.1 to 11.9), while the remaining 127 were more active in HIV- cells (mean TPM = 14.4 vs 7.1; median TPM = 3.4 vs 1.9; fold change range = 1.1 to 19.2; Figure 1, File S2). Of note, even if 6 HML2 loci were expressed in both conditions (TPM values from 1 to 13.4), the HERV loci upregulated in HIV+ cells did not include any member of the HML2 group (File S2). 

### 3.2. Analysis of Individual HERV Loci Modulated in the Presence of HIV Infection

**Figure 3:**
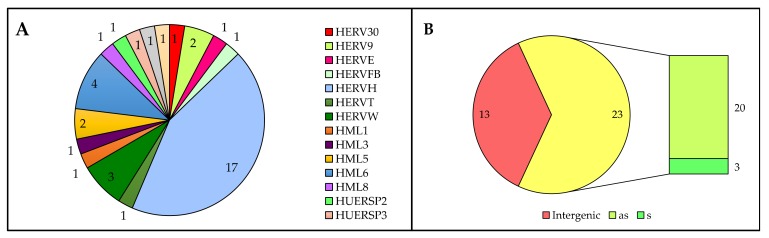
Overview of the individual HERV loci modulated in the presence of HIV infection. (**A**) Number of HERV loci for each group; (**B**) Genomic context of integration: for intragenic HERVs (yellow) the sense (s) or antisense (as) orientation with respect to the surrounding gene is also indicated.

Starting from the above subset of 155 expressed HERVs with minimum TPM ≥ 1 in at least one condition, we identified those HERV loci whose transcriptional activity appeared to be modulated in the presence of HIV infection. In particular, given that a statistically-supported differential expression analysis requires at least 2 samples for each condition [51], we defined as modulated those HERV loci showing at least a 3-fold change in TPM between HIV- and HIV+ cell cultures. This cutoff allowed us to identify 39 HERV loci modulated by HIV infection, being either upregulated (7) or downregulated (32) with respect to uninfected T cells (Figure 1 and Table 1). A total of 17% of the modulated HERV loci belong to the HERV-H group (that is also the one with the highest number of members), followed by HML6 (4%) and HERV-W (3%) (Figure 3). Modulated HERV loci are often integrated within a cellular gene (23 out of 39), and are mostly in antisense orientation (20) (Figure 3). Even if such bias should reduce the possible impact of genic expression on HERV loci activity, it is however possible to observe an apparent HERV transcriptional modulation that is indeed driven by the surrounding gene expression. To evaluate such an effect, we checked if modulated HERV loci and colocalized genes showed a similar pattern of transcriptional variation in the presence of HIV infection (Table 1). Almost all the colocalized genes (20 out of 23) showed TPM values between 0 and 0.8 that were hence lower than the colocalized HERV locus expression values, being mostly not or only slightly modulated in the presence of HIV infection (Table 1). This likely suggests the absence of modulation by the surrounding cellular genes on HERV loci expression. The 3 remaining cellular genes, showing TPM values from 1 to 8.7, had indeed fold changes < 2, while the colocalized HERV loci showed variations from 4 to 9 folds (Table 1). It is noteworthy that the 6 HERV loci with the highest modulation (10- to 19-fold change) were not colocalized with any known gene, suggesting that such HERV expression is independent from cellular gene regulation (Table 1).

### 3.3. Analysis of HERV-Derived Transcripts’ Expression 

In order to evaluate the actual transcriptional activity of the 155 HERV loci with TPM ≥ 1, a de novo reconstruction of HIV+ and HIV- cell transcriptomes was performed with Trinity software [44]. The analysis led to the prediction of 776,844 transcripts that have been aligned back to hg38 reference sequence to identify their genomic origin. Among the inferred transcripts, 0.07% originates from 40 HERV sequences, which represent 26% of the 155 HERVs suggested to be expressed based on the locus TPM value (Supplementary Material, File S3). This result, on the one hand, provides a first indication of the good correlation between read-count based TPM and transcript prediction, while, on the other hand, shows that the presence of reads mapping to a certain genomic sequence is not sufficient itself to assume its transcription into a mRNA. As an additional quality filter, TPM values have been calculated for all the transcripts inferred by Trinity, and the ones matched to HERV loci have been further selected based on a cutoff of TPM ≥ 1 in at least one condition (File S3). In this way, the final set of inferred HERV transcripts included 80 sequences originated by 17 different HERV loci (File S3), among which was a HERV-H locus (HERV ID = 2334) that was already found to be downregulated in the presence of HIV infection (4.3-fold change) (Figure 1 and Table 1). Interestingly, all the 17 HERV loci had high TPM values themselves (from 4 to 610), indicating a good predictive relation between estimated expression of the genomic element and the encoded transcript (File S3). Of further note, 15 of these transcripts assigned to 5 HERV loci have relatively high expression in at least one condition, ranging from 5 to about 64 TPM values, and correspond to 5 of the top-10 expressed HERV loci (TPM from 21 to 610) (File S3), also confirming a direct (even if indicative) relationship between HERV loci with the highest TPM values and transcript abundance. These 15 highly expressed transcripts have been thus visualized in the genomic context, to assess their structure with respect to the originating HERV locus and the eventual colocalized genes (Figure S1). HERVIP locus 4849 (20q13.13), which is the one showing the highest TPM (610 and 365 in uninfected and infected cells, respectively), was associated with 5 transcripts with TPM values from 3.9 to 14.3 (File S3). Of these, two transcript isoforms (DN58338_c3_g1, isoforms 3 and 13) were in antisense orientation with respect to the HERV locus and appeared to be primed by an exon of the colocalized gene ZNFAS1, encoding for a small nucleolar RNA (Figure S1). Of note, ZNFAS1 was expressed at very low levels in both conditions, showing 0,9 TPM without variations in the presence of HIV. Similarly, a third antisense isoform (DN58338_c3_g1_i1, the most expressed of this HERV locus), includes about 2000 nucleotides of the provirus and a downstream Alu element, being apparently primed into the HERV sequence. Another transcript (DN58338_c3_g5_i1) starts from the same position in the opposite direction, hence in the same orientation of the HERV locus, possibly suggesting the presence of a bidirectional promoter (Figure S1). The last transcript of HERVIP 4849 (DN58338_c3_g1_i1) is also in sense orientation, spanning about 2500 nucleotides within the HERV and including at each end an Alu secondary integration (Figure S1). HERV-E locus 4444 is associated with an antisense transcript that seems primed from the HERV 5′LTR and includes an upstream portion of SLFN12L gene intron (Figure S1). Both locus 4696 (HML5, 19q11) and 4224 (HERV-FB, 14q32.31) are associated with a sense transcript showing spliced-like structure that starts upstream of the HERV sequence and includes a small portion of it as a single exon (DN66652_c1_g2_i40 and TRINITY_DN57712_c0_g2_i5, respectively) (Figure S1). While the former transcript starts in a genomic position apparently devoid of cellular genes and other repetitive elements, the latter begins from an exon of the colocalized MOK gene and is in antisense orientation with respect to it, following indeed the HERV locus orientation (Figure S1). Finally, HERV locus 4796 (HML6, 19q13.43) was predicted to produce a transcript showing the highest TPM among HERV-derived transcripts (DN62338_c3_g2_i6, TPM = 63.6 and 41.8 in HIV+ and HIV- cells, respectively), which starts from an exon of the colocalized ZNF8 gene and includes 3 further exons into the HERV locus (Figure S1). The HERV exons mapped between the 5′LTR and *gag* gene, in the *env* gene, and in the 3′LTR. The same transcript has actually been reported in literature, being the product of a naturally-occurring readthrough between the neighboring ZNF8 gene and the HERV locus (ZNF8-ERVK3-1 long non-coding RNA), confirming the reliability of our transcript reconstruction (Figure S1).

### 3.4. Identification of HERV-Derived Transcripts Modulated in the Presence of HIV Infection

Subsequently, we checked which of the selected HERV transcripts were modulated in the presence of HIV infection, setting also in this case a cutoff of at least a 3-fold change of TPM with respect to uninfected cells. We individuated 21 transcripts ascribable to 9 HERV loci: of note, all the identified transcripts were negatively modulated in the presence of HIV infection, showing from 3- to 12-fold decrease in TPM as compared to the uninfected culture (Table 2). In the latter, transcripts were expressed with TPM values from 1 to 8.7 (Table 2). HERV-derived transcripts had a mean length of 500 nucleotides and ranged from a minimum of 229 to a maximum of 1431 nucleotides (Table 2). Alignment with the reference genome sequence showed that almost all transcripts were originated by the sole HERV locus, i.e., without including any chimeric portion with nearby cellular genes or non-coding genomic flanking (Table 2, Figure S2). Two exceptions were the transcripts inferred at HERV-W loci 19q13.2a and 14q32.11, which overlap with an exon of the colocalized genes *ZNF780A* and DGLUCY, respectively (Table 2, Figure S2). Particularly, in both cases, transcript expression seems to be primed by a portion of the cellular gene, and includes a portion of the HERV (3′LTR and *pol* gene, respectively) that is found in antisense orientation, possibly deriving from a readthrough mechanism (Figure S2). As mentioned also in the previous section, a similar situation has been reported for another of the modulated HERV loci, 4796 (HML6, 19q13.43), producing a transcript by a naturally occurring readthrough in the neighboring ZNF8 gene (ZNF8–ERVK3-1), which are however in the same orientation. In our results, the modulated transcripts are found downstream ZNF8–ERVK3-1 exons 6/7, including the 5′ portion of the HML6 locus *env* gene (Figure S1). Concerning the other modulated transcripts, 11 were associated with HERV-E locus 4444 (17q12), showing TPM values from 0.1 to 8.7 (Table 2, Figure S2). One of these transcripts (DN64887_c2_g1_i10) had the highest TPM, showing about a 6-fold increased expression in uninfected cells (Table 2), and was mapped in antisense orientation at the HERV *gag* gene, showing a spliced-like structure (Figure S2). Finally, a HERV-FA locus (2384, 6q25.3) was predicted to encode various transcripts, two of which had TPM values around 2 in HIV- cells (Table 2). Beside these transcripts, a group of different isoforms is worth to be mentioned, suggesting the expression of the whole *env* gene and the downstream 3′LTR (Figure S2, highlighted with red rectangle).

### 3.5. Prediction of HERV-Encoded Putative Proteins

**Figure 4:**
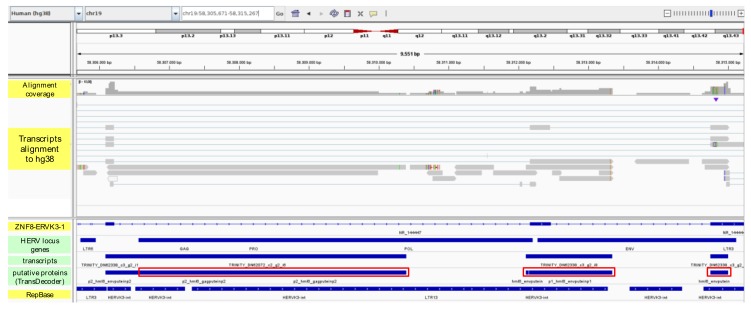
Focus on the puteins encoded by HML6 locus 4796. Puteins and the originating transcripts have been aligned to the locus genomic position, annotated with the retroviral genes as previously identified [43]. The presence of human genes (Gencode version 32) and repetitive elements (RepBase) is also indicated. The puteins overlapping with reported exons are highlighted with a red rectangle.

To complete the present pilot study, peptides and proteins potentially encoded by the transcripts reconstructed by Trinity have been predicted using the software TransDecoder [44]. The obtained amino acid sequences have been compared through BLAT [52] search within a collection of consensus sequences representing the putative proteins (puteins) of each HERV group [9], to identify HERV-derived proteins. Such a search led to the identification of 57 HERV-derived puteins inferred from the transcripts of 23 HERV loci (File S4). These transcripts have then been checked for their expression, and, as already done for HERV loci and predicted transcripts, only the puteins matched with transcripts having TPM values ≥ 1 in at least one condition have been validated for characterization. This filter led to the selection of 8 puteins potentially encoded by 5 HERV loci, as described in Table 3. Importantly, all puteins were correctly matched with the consensus of the group of belonging, suggesting a good predictive value of the pipeline (Table 3). The majority were inferred from transcripts having higher expression in uninfected T cells (5/8) or showing no variations between the two conditions (1/8), except for 2 puteins that were predicted from transcripts with increased expression in HIV+ cells (HML6 4796 DN62338_c3_g2_i6, and HML3 4655 DN62722_c3_g4_i3) (Table 3). Intriguingly, an Env putein was encoded by 3 transcript isoforms of HML6 locus 4796, among which was the already mentioned DN62338_c3_g2_i6, i.e., the ZNF8-ERVK3-1 readthrough transcript showing the highest TPM in both conditions (63.6 and 41.8 in HIV+ and HIV- cells, respectively) (File S3, Figure S1) and being slightly upregulated in HIV-infected cells (1.5-fold change). We further analyzed this HERV locus aligned with the encoded transcripts and putative proteins, observing that, interestingly, the N-terminal portion of Gag and the Env puteins correspond to exons of the ZNF8-ERVK3-1 readthrough transcript, thought to be instead a long non-coding RNA (Figure 4). 

**Figure 5:**
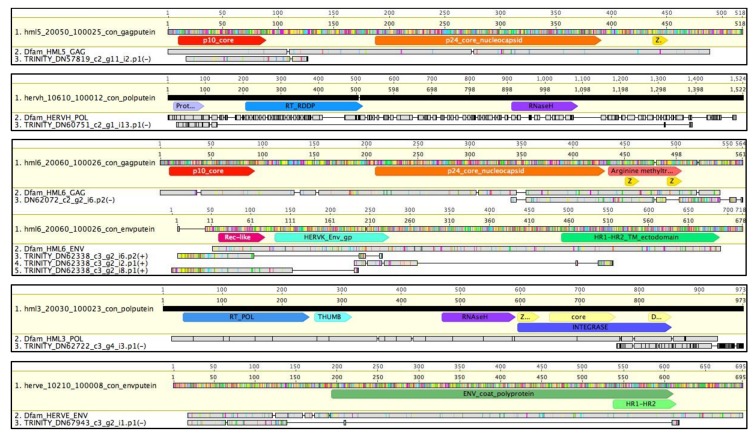
HERV puteins alignment with the respective consensus sequences from Dfam and Vargiu et al. [9]. In the latter, conserved domains have been annotated: dark yellow Z = Zinc finger motif, gp = glycoprotein, HR1-HR2_TM = transmembrane heptad repeats 1 and 2, RT = reverse transcriptase, light yellow z and D = zinc- and DNA-binding domains, respectively. Colored residues indicate divergent amino acids as compared to the reference, which is highlighted by the yellow bar.

The inferred HERV puteins have also been compared with the respective consensus sequences from Dfam [53] and Vargiu et al. [9], and conserved domains have been annotated based on NCBI conserved domains prediction [54] (Figure 5). The analysis showed that the Gag putein encoded by HML6 locus 4796 Gag includes the sole C-terminal half of the protein, presenting a portion of p24 core nucleocapsid domain and one conserved Zinc finger, while a second one has been lost due to a deletion (Figure 5). Similarly, the 4796 HML6 transcript isoforms mapping to the *env* gene encode defective puteins corresponding to a portion of the N-terminal Surface subunit, being predicted to include a Rec-like domain in two cases (Figure 5). Concerning the other puteins, they were inferred by TransDecoder from transcripts with TPM values < 2 and include a Gag putein (HML5 6074 locus), two Pol puteins (HERV-H 3651 and HML3 4655 loci), and an Env putein (HERV-E 4444 locus) (Table 3 and Figure S3). The latter was matched with a Harlequin consensus due to the fact that HERV-E is a major structural contributor of Harlequin mosaic elements, which derive from recombination events [9]. The 6074 HML5 Gag putein corresponded to the core domain, being truncated due to a stop codon at position 126 (Figure 5). Similarly, the 3651 HERV-H Pol putein included the sole Protease domain, lacking the following reverse-transcriptase portion; while 4655 HML3 Pol putein corresponded to the 3′ genic region, presenting hence the C-terminal integrase part only and including the DNA binding domain (Figure 5). Finally, the 4444 HERV-E Env putein corresponded to the first ~140 amino acids of the consensus and did not present any conserved domain (Figure 5).

## 4. Discussion

Currently, the greatest difficulty in studying the expression and biological activity of the HERV loci is related to the high sequence identity, which makes their unambiguous detection through traditional approaches such as PCR or microarray challenging. Such a limitation has led to conflicting information about HERV expression and, in some instances, inconsistent findings, thus representing a confounding factor in the current understanding of HERV physiological and pathological significance [30]. This scenario also includes the analysis of the HERV activity in the context of HIV infection, with different studies reporting either an upregulation of HERV expression in infected individuals or cells (particularly of the HERV-K HML2 group) or, contrarily, the absence of significant differences between the two conditions. 

In more detail, a number of articles showed that HIV infection in patients and cell cultures stimulated the expression of HML2 sequences [22,26,55], even leading to the production of HML2 viral-like particles [26]. Such HIV-mediated upregulation was proposed to rely on the transactivating action of HIV Tat protein, able to increase HML2 transcription in vitro [25]. Accordingly, HML2 expression was reported to be reduced in the presence of effective antiretroviral treatment [23]. Besides HML2 sequences, a role for HIV Tat in the upregulation of HERV expression was also reported for the HERV-W group in blood cells and astrocytes [27]. One of the most comprehensive studies in terms of the number of HERV groups is the one by Vincendeau and colleagues, which used a microarray of 49 representative HERV *pol*-RT sequences derived from 20 major HERV groups to analyze HERV transcription in three persistently HIV-1 infected cell lines and in their uninfected counterparts [56]. Results showed that members of 7 HERV groups, namely HERV-T, HERV-E, HERV-W, ERV9, HML3, HML4 and HML10, were upregulated in productive infected cells [56]. 

For what concerns the opposite scenario, Esqueda and coauthors observed a lack of correlation between HML2 expression and HIV-1 viral load in patients’ plasma, especially after the careful removal of cellular DNA, which allowed accurate evaluation of HML2 RT-PCR signals [28]. Another study reported an absence of variation in HML2 RNA between infected and uninfected women, but observing an increase of HERV-W expression in HIV-infected mothers that was associated with a slight downregulation in HIV-exposed babies as compared to non-exposed ones [29]. 

While the above works employed methodologies unable to distinguish the individual members expressed within a given HERV group, only a few studies analyzed the effect of HIV on the expression of specific HERV loci. In one of these, authors developed a quantitative-PCR protocol able to detect 51 out of the 89 known HML2 proviruses, to measure their specific expression levels in plasma and peripheral blood mononuclear cells from HIV-1-infected patients and uninfected controls [32]. Based on their results, the HML2 loci RNA level was not augmented in the plasma of HIV-1-infected patients, and it was related neither to antiretroviral treatment nor to HIV-1 plasmatic RNA and cellular RNA/DNA levels [32]. A second study revealed that HIV Tat did not broadly activate all the 91 HML2 loci taken into account, but increased the expression of a subset of them (26), while the rest of the proviruses did not show any transcriptional variation or even resulted silenced (12) [31]. Similarly, another recent study productively analyzed HIV-1-infected primary human CD4 T cells for a comprehensive HML2 loci expression profile, confirming absence of significant differences in the overall HML2 expression upon HIV-1 infection and lack of correlation with HIV-1 expression levels [30]. 

In the present study, we aimed to add a piece to this puzzling scenario, trying to overcome the existing difficulties in i) the comprehensive detection of HERV-derived transcripts, ii) their univocal match to the specific HERV locus of origin and, last but not least, iii) the reliable quantification of their expression levels, as an essential requirement to evaluate their possible biological significance. To this purpose, we developed a bioinformatics pipeline that relied on a detailed and exhaustive HERV genomic characterization [9,10,33,34,35], and used it to analyze public transcriptomic data from two human T cell line H9 cultures either uninfected or HIV-1 infected [50], to assess HERV expression in the two conditions and evaluate the effect of HIV modulation. Our approach has multiple advantages with respect to traditional methodologies such as PCR or microarray, which were used in most of previous HERV expression studies. First of all, it allows the univocal match of RNA sequences to the sole genomic portion of origins, given that ambiguous reads, mapping to multiple positions, are discarded before expression analysis. Secondly, read count and expression quantification is carried out at the specific genomic positions of each HERV and cellular gene sequence, avoiding any bias related to nucleotide identity as well as mutation accumulation that can determine cross-reactivity and lack of detection, respectively, which instead affect traditional workflows. Finally, the use of whole transcriptomic data allows us to perform a comprehensive characterization of HERV expression, including the assessment of colocalized gene influence, the reconstruction and quantification of derived transcripts and the prediction of their coding potential. Of course, our study also presents some limitations. The main limitation regards the RNA-seq dataset analyzed [38], which includes a single sample for each of the two conditions (HIV+ and HIV-), preventing the use of differential expression analyses to identify modulated HERV loci. In fact, public RNA-seq data including HIV+ patients/primary cells and uninfected controls are not so common yet, because HIV infection is mainly analyzed through the sequencing of HIV genome/products only (e.g., through real-time PCR), given that integrations are not associated with specific positions as in the case of HERV loci. In addition, the few RNA-seq datasets dedicated to HIV infection are focused on the alteration of cellular gene expression, and hence have technical characteristics (such as single end reads of 50 bp, ~20 million reads/sample) that make them useless for the analysis of HERV expression. This limit is due to the fact that repetitive elements are present in multiple copies sharing high nucleotide identity, and longer paired-end reads in higher quantity are needed to allow the detection and univocal mapping of HERV expression. These characteristics were found only in the RNA-seq dataset from Gupta et al., having high sequencing depth (≈80 million reads/sample) combined with high quality paired-end reads (150+150 bp, mean fragment size >350) [38]. As a second limitation of our study, the analyzed RNA-seq data includes a single time point of HIV infection (7 days post infection) [38]. Even if this time is surely representative of the effects of HIV on cellular transcriptome, as confirmed by Gupta et al. through the analysis of protein-coding cellular genes known to be strongly upregulated in HIV-1 infection [38], it remains a single point of view that does not allow us to evaluate changes in HERV expression according to the different phases of HIV infection and antiviral immune responses. Given this, the here reported characterization of HERV loci transcriptional activity in the presence of HIV infection provides a preliminary but very comprehensive overview, worthy of further confirmation in subsequent experimental studies. These are needed to confirm the expression of the identified HERV loci and to assess the transcriptional variations associated with the different phases of both viral life cycle and immune response in a wider biological sample. 

Using our bioinformatics pipeline, we found that around 5% of the 3251 investigated HERV loci was expressed in at least one condition, being generally downregulated in HIV-infected cells. In fact, 82.5% of expressed HERV loci showed higher TPM in control-uninfected cultures, with a mean TPM of 11.3 against 6.3 in HIV+ cells (File S2). This result is in contrast with the hypothesis that HIV can exert a generalized activation of HERV expression. Intriguingly, downregulated HERVs in HIV+ cells included all the 6 expressed HML2 loci, confuting the transactivation of the group by HIV proteins. In particular, among the few studies assessing HERV expression at the locus level, Young and coworkers identified 3 HML2 proviruses (6q25.1, 8q24.3, and 19q13.42) that were upregulated in HIV-1-infected cells, while a single HML2 locus (12q24.33) was found to be repressed in the presence of active HIV replication [30]. While we did not find positive modulation for any HML2 locus in the presence of HIV infection, we can confirm that HML2 locus 3922 (12q24.33) was actually downregulated in HIV+ cells, showing a 2-fold TPM decrease (File S2). Another recent study analyzed RNA-seq profiles from SupT1 cells expressing HIV Rev vs control cells, using a classical bioinformatics pipeline similar to the one employed in this study but limited to the analysis of HML2 loci expression, and without transcriptome analysis [57]. Based on our knowledge, this is the only other study on HERV expression in RNA-seq data from HIV-infected cells. Authors showed that 9 out of 94 HML2 loci (1q21.3, 3q12.3, 4p16.1b, 6p21.1, 6q25.1, 11q12.3, 19q13.12b, 20q11.22 and 22q11.23) were expressed in all samples, and proviral mRNA from three of them (3q12.3, 4p16.1b and 22q11.23) accumulated in the cytoplasm of Rev-expressing cells [57]. As compared to their results, we also found that HML2 loci 6062 (1q21.3) and 864 (19q13.12b) are expressed in both conditions, with slightly increased TPM in HIV- cells (File S1), even if discrepancies between our and their HERV expression profiles can be expected due to the differences in the experimental design (different cell line, HIV infection vs HIV Rev transduction, RNA extraction 7 days vs 2 days post infection). 

To better evaluate the observed general downregulation of HERV expression in the presence of HIV infection, we introduced an expression cutoff considering only those HERV loci showing TPM ≥ 1 in at least one condition. The remaining 155 HERV loci confirmed such a general behavior, being downregulated in HIV+ cells in 81.9% of cases (Figure 1, File S2). These first results i) confirm that a certain level of “basal” HERV expression is a physiological phenomenon, and should be considered when investigating HERV upregulation in a diseased context to avoid overinterpreted conclusions; and ii) suggest that it is unlikely that HIV infection can lead to an unspecific upregulation of HML2 sequences or to the general activation of any other HERV group. In fact, HERV expression was shown in members of 30 different HERV groups, with percentages of expressed members ranging from 2.5% to 23% (Figure 2). This indicates that approaches estimating the expression of whole HERV groups, i.e., using broad-spectrum primers, just catch a partial picture of their transcriptional activity, being biased towards the variable proportion of members that are actually expressed. 

Another important advantage in RNA-seq analysis of HERV loci expression is the possibility to consider their genomic context of integration, which is able to greatly influence HERV transcriptional activity and its variation. Indeed, the different pathological conditions, including viral infections, can lead to the dysregulation of certain cellular genes and, as a consequence, of an eventual subset of HERV loci colocalized with them. The same can occur with some therapeutic treatments, especially if linked to DNA alterations [58]. Such an indirect modulation, when present, has the potential to shape HERV transcriptome, as confirmed by the fact that 77% of the expressed HERV and approximately 60% of the HERV loci modulated in the presence of HIV infection loci were inserted into human genes (Table 1 and Figure 3). For this reason, we checked the expression of HERV-colocalized genes, finding that in the majority of cases (88.5%) their presence is unlikely to have an effect on HERV loci expression and modulation by HIV infection (Table 1). Of note, expression studies based on the sequencing of the whole transcriptome allows us to perform transcript reconstruction and protein prediction, linking the presence and quantification of reads to the concrete possibility that such expressed sequences can produce a transcript with coding potential. To this purpose, we used Trinity software to infer de novo the whole cellular transcriptome, identifying ~560 transcripts deriving from 40 individual HERV loci (File S3). Among these, the 15 top-expressed transcripts (TPM 5 to 64) originated from 5 of the top-10 expressed HERV loci (TPM 21 to 610), confirming a good relationship between reads and transcript abundance when a certain threshold of expression is reached. As seen with HERV loci activity, transcript expression was also largely higher in uninfected cells (Figure 1 and Table 1), and 21 HERV transcripts were shown to be modulated in HIV+ cells, being all downregulated (Table 2). Overall, these results further confirm the absence of general upregulation of HERV expression by HIV. Interestingly, HERV transcript characterization highlighted isoforms possibly produced by a readthrough mechanism that occurred between the HERV locus and exons of a colocalized cellular gene. This was the case of a transcript linked to 4796 HML6 locus (DN62338_c3_g2_i6), already known as ZNF8-ERVK3-1 and originated by natural readthrough from ZNF8 gene (Figure S1). This transcript was the most expressed among the HERV-derived transcripts, showing the highest TPM in HIV+ cells (63.6, File S3) and, contrary to its description as long non-coding RNA, it was predicted to encode partial Gag and Env puteins in correspondence of the inferred exons (Table 3 and Figure 4). The same HML6 locus was reported to encode another small transcript (ERVK3-1, ENSG00000142396) found in various healthy tissues, leading to the hypothesis of an extensive HML6 activity [59]. In our recent characterization study of the HML6 group, we provided the first description of a Rec domain within the *env* sequence of 23 HML6 elements, including 4796 HML6 locus and ERVK3-1 transcript [35]. This Rec domain has also been recognized in the putative Env proteins encoded by 4796 HML6 transcripts, further confirming its presence (Figure 4). This, together with the fact that 4796 HML6 locus and DN62338_c3_g2_i6 ZNF8-ERVK3-1 transcript were both upregulated in HIV+ infected cells with high TPM values (28.2 and 63.6, respectively), makes them worthy of further investigation in the context of HERV interplay with HIV, due to their possible biological significance. Similarly, a protein coding capacity has been predicted for transcripts associated with 23 HERV loci overall, resulting in a final set of 8 puteins encoded by 5 HERV loci selected based on TPM cutoff (Table 3, Figure 4 and Figure S3). The identified puteins correspond to Gag (2), Pro-Pol (2) and Env (4) proteins and, even if defective, included some recognizable, conserved domains. Further studies are needed to assess the biological activity of HERV expression products in a wider biological sample, to identify specific HERV loci differentially expressed in the presence of HIV infection and to study their interplay with viral and immune system genes and proteins.

## 5. Conclusions

The comprehensive characterization of individual HERV loci expression in human H9 cells revealed a proportion of elements with transcriptional activity, which were mostly downregulated in the presence of HIV infection. This result, on the one side, confutes the hypothesis that HERVs, and especially HML2 elements, are specifically activated by HIV, making it unlikely that they can directly sustain HIV infection, i.e., complementing HIV-defective proteins or taking part in drug resistance. On the other hand, our study confirms that HERV expression is a physiological phenomenon, whose contribution to the human transcriptome and proteome is still far from being clarified. It is probable that HIV infection and/or the subsequent activation of antiviral immune pathways might exert a negative effect on HERV transcription. Indeed, the evidence of different patterns of expression within the same HERV group, as well as the presence of a minority of HERV loci whose expression was instead increased in HIV+ cells, leaves open the possibility that a few HERV sequences can be specifically upregulated by HIV presence, asking for further studies to investigate the molecular mechanisms underlying their activation. To this purpose, analyses of whole transcriptomic data appear the most suitable approach to specifically and exhaustively characterize HERV expression and its modulation in an appropriate biological sample.

## Figures and Tables

**Table 1:** Individual HERV loci modulated in the presence of HIV infection.

ID ^1^	Group	TPM HIV-	TPM HIV+	FC	Coordinates (hg38)	St.	Colocalized Genes ^2^	TPM HIV-	TPM HIV+	FC
*HERV loci upregulated in HIV+ cells*										
4594	HERVH	0.12	1.45	11.9	19:5548576-5553212	+				
3166	HERVH	0.12	1.07	8.9	9:131547214-131551936	+	PRRT1B (+)	0.04	0.21	4.9
2307	HERV30	0.35	1.90	5.4	6:118617102-118626802	+	CEP85L (-)	0.34	0.49	1.4
2082	HERV9	0.25	1.38	5.4	6:16455730-16464625	+	ATXN1 (-)	0.04	0.03	1.2
4457	HML6	0.31	1.56	4.9	17:43872528-43874324	+				
1744	HERVH	2.91	10.11	3.5	5:702471-708501	+				
3112	PABL	0.45	1.32	3.0	9:94265146-94270221	-	ZNF169 (+)	0.01	0.01	-
*HERV loci downregulated in HIV+ cells*										
691	HML6	1.97	0.10	19.2	2:136071818-136077261	-				
5407	HML6	2.29	0.17	13.1	X:57102981-57109396	+				
2658	HERVH	2.52	0.21	12.1	7:143105117-143110504	+				
4741	HERVH	22.77	2.16	10.5	19:46102204-46109974	-				
2476	HML3	3.21	0.32	10.1	7:43859699-43864985	-				
3788	HERVH	3.39	0.39	8.8	12:48111344-48115676	-	PFKM (+)	8.66	4.88	1.8
6q27b	HERVW	4.33	0.55	7.9	6:166891083-166898253	+	AL159163.1 (-)	0.00	0.00	-
4511	MER57	1.07	0.15	7.1	18:13700682-13708050	+	FAM210A (-)	0.11	0.07	1.5
5387	HML6	41.38	6.53	6.3	X:53159114-53163826	-				
3637	HUERSP2	2.23	0.35	6.3	11:108198480-108204806	+				
933	HERVH	3.55	0.58	6.1	3:44344658-44350393	-	TCAIM (+)	0.28	0.17	1.6
957	HERV9	1.78	0.29	6.1	3:51742261-51747980	+				
5815	HERVFB	39.41	6.51	6.1	1:8921052-8927320	+				
4329	HERVH	1.56	0.29	5.4	15:101072487-101078293	+	LRRK1 (+)	0.18	0.17	1.08
3436	HUERSP3	5.73	1.16	5.0	11:34897477-34907140	+	APIP (-)	0.68	0.52	1.3
1113	HERVH	3.24	0.70	4.6	3:128959818-128965400	+	KIAA1257 (-)	1.83	1.46	1.3
4374	HERVT	13.98	3.07	4.5	16:35267618-35277076	-				
1407	HERVH	8.37	1.84	4.5	4:54020079-54025546	+	CHIC2 (-)	0.05	0.00	-
5356	HML8	2.70	0.59	4.5	X:46486517-46490287	-	KRBOX4 (+)	0.07	0.06	1.2
2334	HERVH	18.93	4.45	4.3	6:130192311-130198222	+	SAMD3 (-)	0.23	0.16	1.4
3887	HML5	1.85	0.46	4.0	12:100159365-100165461	-	GOLGA2P5 (-)	0.78	0.23	3.5
18p11.21	HERVW	1.95	0.48	4.0	18:4681680-4692409	+				
Xp11.21	HERVW	2.47	0.63	3.9	X:7699169-7704068	-				
2219	HML1	5.49	1.45	3.8	6:78854118-78858747	+				
1549	HML5	1.75	0.46	3.8	4:106109837-106114668	+	TBCK (-)	2.58	1.56	1.7
4475	HERVH	1.16	0.32	3.6	17:55467773-55476512	+	SMIM36 (-)	0.00	0.00	-
3370	HERVH	4.52	1.29	3.5	10:120844932-120850561	+	WDR11-AS1 (-)	0.29	0.14	2.1
1975	HERVH	14.08	4.34	3.2	5:124966686-124972226	-	LINC02240 (+)	0.01	0.01	-
3701	HERVH	8.68	2.68	3.2	12:9962437-9968690	-	CLEC12A (+)	0.20	0.13	1.6
5705	HERVH	7.88	2.53	3.1	X:131712025-131717764	+	FIRRE (-)	0.00	0.00	-
3544	HERVE	2.09	0.69	3.0	11:71886034-71890084	+	AP002495.1 (-)	0.04	0.00	-
4610	HERVH	1.96	0.64	3.0	19:9635997-9642066	-	ZNF561-AS1 (+)	0.21	0.54	2.6

^1^ ID referred to Vargiu et al. 2016, except for HERV-W locus names that come from Grandi et al. 2016. ^2^ Based on Genecode annotations (version 32). FC = Fold Change, St. = Strand. TPM values are represented with a color gradient from red (highest) to green (lowest).

**Table 2:** HERV-derived transcripts modulated in the presence of HIV infection.

HERV Locus Information					Transcript Description			Transcript in HIV- Cells		Transcript in HIV+ Cells	
ID ^1^	Group	Coloc. Genes	TPM HIV-	TPM HIV+	Trinity ID	nt	FC	read n°	TPM	read n°	TPM
**19q13.2a**	HERV-W	ZNF780A	83.78	94.62	DN62359_c2_g9_i3 *	378	**3.6**	18	1.29	5	0.35
**4444**	HERV-E	SLFN12L	214.46	74.94	DN67670_c4_g2_i6	420	**3.3**	77	4.56	22	1.37
					DN67943_c3_g5_i5	512	**3.3**	91	3.84	26	1.15
					DN67943_c3_g2_i1	889	**3.1**	106	1.93	33	0.63
					DN59238_c1_g1_i11	264	**3.5**	15	2.18	4	0.62
					DN60987_c8_g9_i1	442	**3.1**	75	4.07	23	1.31
					DN67799_c2_g4_i2	431	**3.8**	23	1.29	6	0.34
					DN61824_c3_g12_i1	372	**3.5**	60	4.39	16	1.24
					DN67943_c3_g5_i8	629	**5.1**	60	1.82	11	0.36
					DN57253_c4_g1_i1	257	**12.3**	13	2.01	1	0.16
					DN64887_c2_g1_i10	339	**5.7**	99	8.71	16	1.52
					DN67670_c4_g1_i22	1025	**4.4**	112	1.69	24	0.38
**2384**	HERV-FA	TMEM181	124.57	58.95	DN59786_c3_g6_i2	260	**6.1**	13	1.96	2	0.32
					DN59236_c4_g1_i1	229	**8.5**	9	1.81	1	0.21
**4796**	HML6	ZNF8 - ERVK3-1	22.68	28.20	DN62338_c3_g2_i2	838	**9.5**	58	1.14	6	0.12
**4618**	HERVE	ZNF700	23.27	8.57	DN62411_c1_g3_i5	513	**4.5**	24	1.01	5	0.22
**3651**	HERVH	DSCAML1	31.76	14.32	DN60751_c2_g1_i13	340	**4.6**	13	1.14	3	0.25
**14q32.11**	HERVW	DGLUCY	14.06	7.88	DN64353_c11_g2_i6 *	1431	**3.3**	157	1.54	45	0.47
**3955**	HERVH	-	6.18	4.33	DN61830_c1_g1_i9	324	**3.7**	16	1.49	4	0.40
**2334**	HERVH	SAMD3	18.93	4.45	DN67393_c0_g6_i1	229	**4.7**	10	2.01	2	0.43
					DN60366_c3_g1_i11	369	**5.4**	17	1.27	3	0.24

^1^ ID referred to Vargiu et al. 2016, except for HERV-W locus names that come from Grandi et al. 2016. * The transcript includes an exonic sequence portion from the colocalized gene. FC = Fold Change, St. = Strand. TPM values are represented with a color gradient from red (highest) to green (lowest).

**Table 3:** HERV-derived putative proteins.

HERV Locus ID^1^	HERV Group	Colocalized Gene	Transcript ID	HIV- TPM	HIV+ TPM	Protein Match	Q Size	Q Start	Q End
6074	HML5	-	DN57819_c2_g11_i2	0.95	0.56	hml5_gagputein	108	0	96
3651	HERV-H	DSCAML1	DN60751_c2_g1_i13	1.14	0.25	HERVH_ polputein	112	25	108
4796	HML6	ZNF8 - ERVK3-1	DN62072_c2_g2_i6	2.42	1.68	hml6_gagputein	188	6	134
			DN62338_c3_g2_i2	1.14	0.12	hml6_envputein	99	1	35
			DN62338_c3_g2_i6	41.79	63.59	hml6_envputein	109	49	97
			DN62338_c3_g2_i8	2.29	2.30	hml6_envputein	157	56	157
4655	HML3	ZNF493	DN62722_c3_g4_i3	0.12	0.99	hml3_polputein	193	6	155
4444	HERV-E	SLFN12L	DN67943_c3_g2_i1	1.93	0.63	harlequin_envputein	132	0	107

^1^ID referred to Vargiu et al. 2016. Q = query. TPM values are represented with a color gradient from red (highest) to green (lowest).

## Supplementary Materials

The following are available online at https://www.mdpi.com/1999-4915/12/4/481/s1, File S1: detailed description of the bioinformatics pipeline for expression analyses. File S2: HERV loci expression in HIV-infected and uninfected T cells, File S3: HERV-derived transcript expression in HIV-infected and uninfected T cells. File S4: HERV-derived putative proteins inferred from reconstructed transcriptome, Figure S1: integrated genomic view of the 15 top-expressed HERV transcripts with the originating HERV loci and eventual colocalized genes and repetitive elements, Figure S2: integrated genomic view of HERV transcripts modulated in the presence of HIV infection with the originating HERV loci and eventual colocalized genes and repetitive elements, Figure S3: integrated genomic view of HERV putative proteins inferred from HERV-derived transcripts with the originating HERV loci and eventual colocalized genes and repetitive elements.
