# Supplementary material for: Comprehensive Analysis of HERV Transcriptome in HIV+ Cells: Absence of HML2 Activation and General Downregulation of Individual HERV Loci
Source: Viruses. 2020 Apr 23;12(4):481. doi: 10.3390/v12040481 (PMC7232394; doi:10.3390/v12040481)
Supplement: Supplementary file 1 [file viruses-12-00481-s001.zip › updated_suppl_files/File S1.docx]

**File S1:**

**bioinformatics pipeline for HERV loci and transcript expression analyses**

**Background: HERV and gene reference datasets**

In general, the analysis of HERV and cellular gene expression in RNA-seq data requires an exact knowledge of the univocal genomic position of each element within the human genome. For cellular genes, we used as a reference the Gencode dataset (version 29) [1] that was downloaded as .gtf annotation file ([https://www.gencodegenes.org](https://www.gencodegenes.org/)) and included the genomic coordinates of 58721 genic elements. For HERV loci, we relied on a comprehensive database that we developed, including about 3170 individual HERV loci classified according to their nucleotide structure and phylogeny into 39 canonical HERV groups plus 31 so-called non canonical groups. The members of the latter showed various degree of mosaicism and were classified according to their composition. Such a HERV dataset was developed starting from the identification and classification of the most integer HERV integrations by the software RetroTector [2], which scans the genome searching for conserved retroviral motifs and, after evaluating their distance and position, reconstructs the retroviral chain and the genes’ open reading frames (ORFs) [3]. Hence, the members of this dataset should represent the most conserved HERV integration, i.e. the ones more prone to retain transcriptional activity. In addition, for sake of completeness, the dataset has been integrated with the information deriving from the dedicated characterization of individual HERV groups relevant for human physiology and/or investigated for their transcriptional activity in human disorders. Particularly, we included all the HERV loci identified in the exhaustive characterization of HERV-W [4], HERV-K(HML-10) [5], HERV-K(HML6) [6], some of which were missing in the starting RetroTector dataset due to their lower degree of completeness. In this way, we ended up with a HERV database including the genomic coordinates, classification and structural information of 3251 HERV loci.

The information for cellular genes and HERV loci (including chromosome, start, end, strand and ID) were included in respective ﻿gene transfer format (gtf) files for subsequent analyses.

**Step 1. Reads’ mapping and counting**

**Mapping to hg38**

.fastq read files have been mapped to the assembled reference sequence of the human genome (GRCh38/hg38, referred as human reference genome in the following text) downloaded as ﻿annotation GTF file from NCBI Genome Browser ([https://hgdownload.soe.ucsc.edu/downloads](https://hgdownload.soe.ucsc.edu/downloads.html#human)). Alignment to the human genome has been performed with STAR RNA-seq aligner [7], version 2.5.2a (﻿<https://github.com/alexdobin/STAR/releases>) following the basic protocol for read alignment to a reference genome [8]. Unmapped reads as well as reads mapping to multiple positions have been discarded prior to subsequent analyses.

**Read count at HERV and gene positions**

As described in details above, we took into account 3251 univocal HERV sequences [2,6,9–11] and 58721 cellular genes from Gencode collection [1], version 29. Raw counts of the number of reads mapping at each HERV and cellular gene genomic coordinates (provided as .gtf file based on the above reference databases) have been quantified through htseq-count, a tool developed within Python-3.6.0 HTSeq framework that preprocesses RNA-Seq data for expression analysis by counting the overlap of reads with given genomic positions [12] ([https://pypi.org/project/HTSeq/](https://pypi.org/project/HTSeq/#files)). Raw reads’ count have been exported as .gtf files for further analyses

**Step 2. Raw counts expression analysis**

**Quantification of HERV and gene expression**

Raw read counts of HERV loci and cellular genes have been imported in RStudio (version 1.2.1335), an integrated development environment for the R programming language [13] ([https://rstudio.com](https://rstudio.com/)). After the creation of a matrix of HERV loci and cellular genes’ read counts, the DESeq2 package [14] (<http://www.bioconductor.org/packages/release/bioc/html/DESeq2.html>) has been used to estimate each HERV/gene expression value in the two conditions. Particularly, the relative abundance of reads has been calculated as transcripts per million (TPM), a quantification that take normalize the number of reads based on the length of the corresponding HERV/gene (as obtained by the difference between end and start genomic positions). This allowed to compare expression levels obtained from genomic elements with variable lengths (from <1 Kb to tenths of Kb). For subsequent analyses, we set a threshold of minimum expression of TPM ≥ 1 in at least one condition.

**Individuation of HERVs and genes modulated in HIV+ cells**

TPM expression values of each HERV locus and cellular gene have been compared between HIV+ and HIV- conditions. Particularly, given that differential expression analysis could not be run due to the absence of biological replicates, we defined as “modulated” those HERVs/genes showing at least 3 folds-change in TPM values. Accordingly, HERV/genes upregulated or downregulated in HIV+ cells were the ones showing at least 3 folds increase or decrease (respectively) of their TPM values as compared to HIV- cells.

**Step 3. Transcriptome reconstruction and expression analysis**

***De novo* reconstruction of HIV+ and HIV- cells’ transcriptome**

Cellular transcriptome has been inferred *de novo* without the guide of a reference genome sequence from .fastq read files using Trinity software (version 2.5.1) [15,16] (<https://github.com/trinityrnaseq/trinityrnaseq/releases>). The obtained transcripts, as included in Trinity transcriptome fasta file, have been mapped to the human reference genome (in fasta format, as downloaded from <ftp://ftp.ensembl.org/pub/release-99/fasta/homo_sapiens/dna/>) with GMAP aligner [17] (<http://research-pub.gene.com/gmap/>). The latter is optimized for the alignment of mRNA-derived cDNA sequences ﻿and generates accurate gene structures with splice sites identification [17]. The alignment of the transcriptome to the human reference genome has been visualized in the Integrative Genomics Viewer (IGV) [18] (<http://software.broadinstitute.org/software/igv/>) together with annotations for human genes (Gencode, version 29 [1]) and HERVs (RepeatMasker annotations [19,20] plus annotations for each HERV locus included in our database). The visualization of transcripts in the genome context with the above annotations allowed us to evaluate Trinity transcripts’ structure as compared to gene exons or – in the case of the 3251 HERV loci – in relation to the individual viral genes of each HERV integration as characterized in our previous works [2,6,9,10].

**Quantification of HERV and gene expression**

The relative abundance of Trinity transcripts in the two conditions has been estimated through Salmon, a specific tool for quantification of transcript inferred from RNA-seq data [21] (<https://salmon.readthedocs.io/en/latest/index.html>). In particular, we used Salmon mapping-based quantification against previously generated transcriptome indices [22].

**Individuation of HERV transcripts modulated in HIV+ cells**

The expression levels of HERV-derived transcripts reaching a threshold of minimum expression of TPM ≥ 1 in at least one condition have been compared between the two conditions. As already mentioned for HERV loci TPM, given that differential expression analysis could not be run due to the absence of biological replicates, we defined as “modulated” those HERV transcripts showing at least 3 folds-change in TPM values. Accordingly, HERV transcripts upregulated or downregulated in HIV+ cells were the ones showing at least 3 folds increase or decrease (respectively) of their TPM values as compared to HIV- cells.

**Step 4. Putative proteins’ prediction**

The protein coding potential of Trinity transcripts has been investigated using the software TransDecoder, version 5.5.0 (<https://github.com/TransDecoder/TransDecoder/releases>). Briefly, TransDecoder infers likely coding regions within transcript sequences, identifying as first step the long open reading frames (ORFs) and subsequently ranking them based on their coding capacity, according to sequence composition and codon frequencies. Finally, TransDecoder predicts a list of putative peptides, providing information about completeness (presence of start and a stop codon), strand, and coordinates of the ORF in the transcript sequence. More in detail, coding sequences identification in our transcripts by TransDecoder has been based on the following criteria: i) a minimum length of the ORF corresponding to at least 100 amino acids, ii) a log-likelihood coding score > 0, which is greatest when the ORF is scored in the 1st reading frame as compared to scores in the other 2 forward reading frames, and iii) a PSSM to refine the start codon prediction. In addition, to maximize sensitivity for capturing ORFs that may have functional significance in HERV transcripts, TransDecoder ORFs have been searched for homology to a list of consensus proteins from all HERV groups [2] by BLAT search [23] with default parameters (minimum score = 30, minimum identity= 25%).

**Bibliography**

1. Harrow, J.; Frankish, A.; Gonzalez, J.M.; Tapanari, E.; Diekhans, M.; Kokocinski, F.; Aken, B.L.; Barrell, D.; Zadissa, A.; Searle, S.; et al. GENCODE: the reference human genome annotation for The ENCODE Project. *Genome Res.* **2012**, *22*, 1760–74.

2. Vargiu, L.; Rodriguez-Tomé, P.; Sperber, G.O.; Cadeddu, M.; Grandi, N.; Blikstad, V.; Tramontano, E.; Blomberg, J. Classification and characterization of human endogenous retroviruses mosaic forms are common. *Retrovirology* **2016**, *13*.

3. Sperber, G.; Airola, T.; Jern, P.; Blomberg, J. Automated recognition of retroviral sequences in genomic data - RetroTector©. *Nucleic Acids Res.* **2007**, *35*, 4964–4976.

4. Grandi, N.; Cadeddu, M.; Blomberg, J.; Tramontano, E. Contribution of type W human endogenous retrovirus to the human genome: characterization of HERV-W proviral insertions and processed pseudogenes. *Retrovirology* **2016**, *13*, 1–25.

5. Grandi, N.; Cadeddu, M.; Pisano, M.P.; Esposito, F.; Blomberg, J.; Tramontano, E. Identification of a novel HERV-K(HML10): Comprehensive characterization and comparative analysis in non-human primates provide insights about HML10 proviruses structure and diffusion. *Mob. DNA* **2017**, *8*.

6. Pisano, M.P.; Grandi, N.; Cadeddu, M.; Blomberg, J.; Tramontano, E. Comprehensive characterization of the HERV-K(HML-6): overview of their structure, phylogeny and contribution to the human genome. *J. Virol.* **2019**, *93*, e00110-19.

7. Dobin, A.; Davis, C.A.; Schlesinger, F.; Drenkow, J.; Zaleski, C.; Jha, S.; Batut, P.; Chaisson, M.; Gingeras, T.R. STAR: Ultrafast universal RNA-seq aligner. *Bioinformatics* **2013**.

8. Dobin, A.; Gingeras, T.R.; Spring, C. Mapping RNA-seq Reads with STAR. *Curr Protoc Bioinforma.* **2016**, *51*, 1–19.

9. Grandi, N.; Cadeddu, M.; Blomberg, J.; Tramontano, E. Contribution of type W human endogenous retroviruses to the human genome: Characterization of HERV-W proviral insertions and processed pseudogenes. *Retrovirology* **2016**.

10. Grandi, N.; Cadeddu, M.; Pisano, M.P.; Esposito, F.; Blomberg, J.; Tramontano, E. Identification of a novel HERV-K(HML10): comprehensive characterization and comparative analysis in non-human primates provide insights about HML10 proviruses structure and diffusion. *Mob. DNA* **2017**, *8*, 15.

11. Subramanian, R.P.; Wildschutte, J.H.; Russo, C.; Coffin, J.M. Identification, characterization, and comparative genomic distribution of the HERV-K (HML-2) group of human endogenous retroviruses. *Retrovirology* **2011**, *8*, 90.

12. Anders, S.; Pyl, P.T.; Huber, W. HTSeq-A Python framework to work with high-throughput sequencing data. *Bioinformatics* **2015**.

13. Rstudio Team RStudio: Integrated development for R. RStudio, Inc., Boston MA. *RStudio* 2016.

14. Love, M.I.; Huber, W.; Anders, S. Moderated estimation of fold change and dispersion for RNA-seq data with DESeq2. *Genome Biol.* **2014**, *15*, 550.

15. Grabherr, M.G.; Haas, B.J.; Yassour, M.; Levin, J.Z.; Thompson, D.A.; Amit, I.; Adiconis, X.; Fan, L.; Raychowdhury, R.; Zeng, Q.; et al. Full-length transcriptome assembly from RNA-Seq data without a reference genome. *Nat. Biotechnol.* **2011**.

16. Haas, B.J.; Papanicolaou, A.; Yassour, M.; Grabherr, M.; Blood, P.D.; Bowden, J.; Couger, M.B.; Eccles, D.; Li, B.; Lieber, M.; et al. De novo transcript sequence reconstruction from RNA-seq using the Trinity platform for reference generation and analysis. *Nat. Protoc.* **2013**, *8*, 1494–1512.

17. Wu, T.D.; Watanabe, C.K. GMAP: A genomic mapping and alignment program for mRNA and EST sequences. *Bioinformatics* **2005**.

18. Thorvaldsdóttir, H.; Robinson, J.T.; Mesirov, J.P. Integrative Genomics Viewer (IGV): High-performance genomics data visualization and exploration. *Brief. Bioinform.* **2013**.

19. Smit, A.; Hubley, R.; Green, P. RepeatMasker Open-4.0. 2013-2015 . Available online: http://repeatmasker.org.

20. Bao, W.; Kojima, K.K.; Kohany, O. Repbase Update, a database of repetitive elements in eukaryotic genomes. *Mob. DNA* **2015**, *6*, 11.

21. Patro, R.; Duggal, G.; Love, M.I.; Irizarry, R.A.; Kingsford, C. Salmon: fast and bias-aware quantification of transcript expression using dual-phase inference. *Nat. Methods* **2017**.

22. Srivastava, A.; Sarkar, H.; Gupta, N.; Patro, R. RapMap : a rapid , sensitive and accurate tool for mapping RNA-seq reads to transcriptomes. **2016**, 192–200.

23. Kent, W.J. BLAT - The BLAST-like alignment tool. *Genome Res.* **2002**, *12*, 656–664.
