# Supplementary figures and images for: Comprehensive Analysis of HERV Transcriptome in HIV+ Cells: Absence of HML2 Activation and General Downregulation of Individual HERV Loci
Source: Viruses. 2020 Apr 23;12(4):481. doi: 10.3390/v12040481 (PMC7232394; doi:10.3390/v12040481)

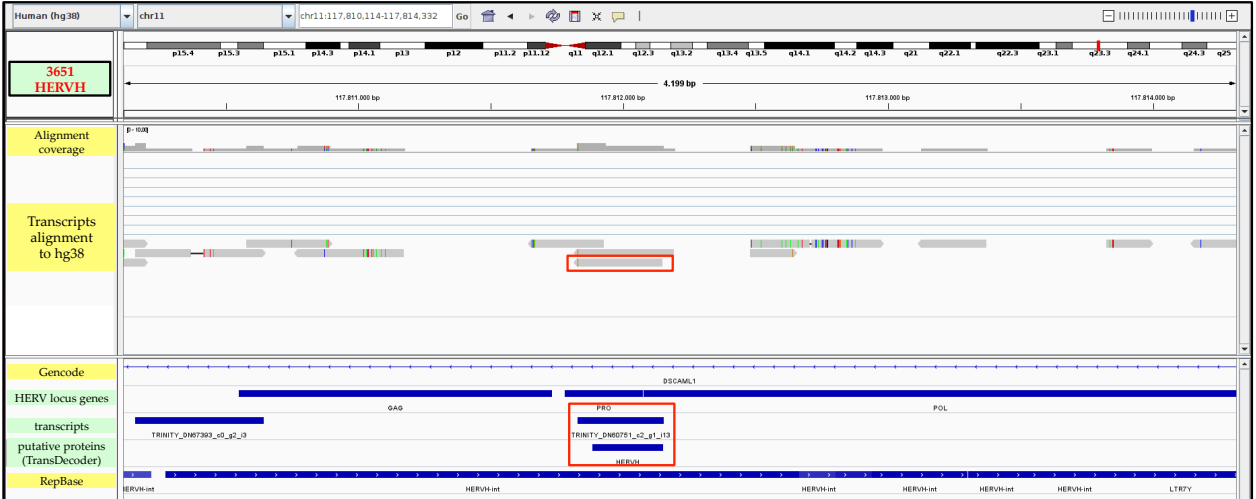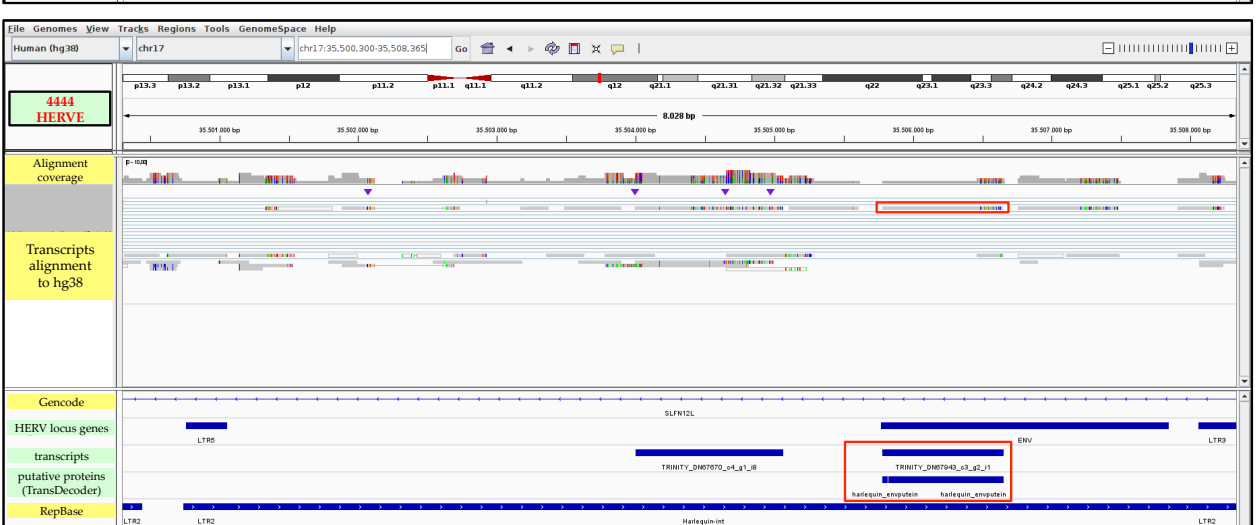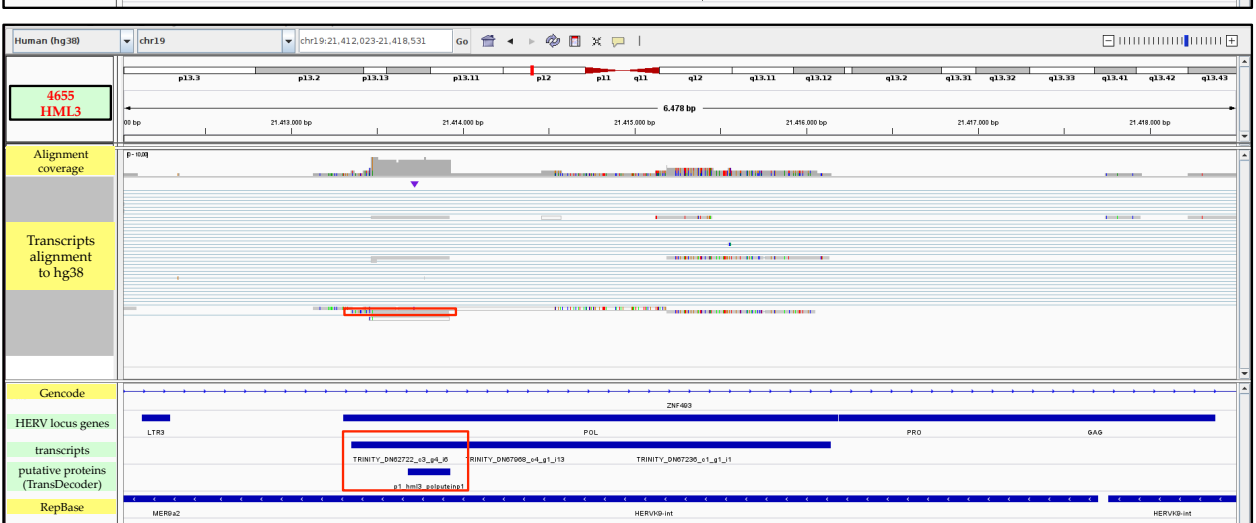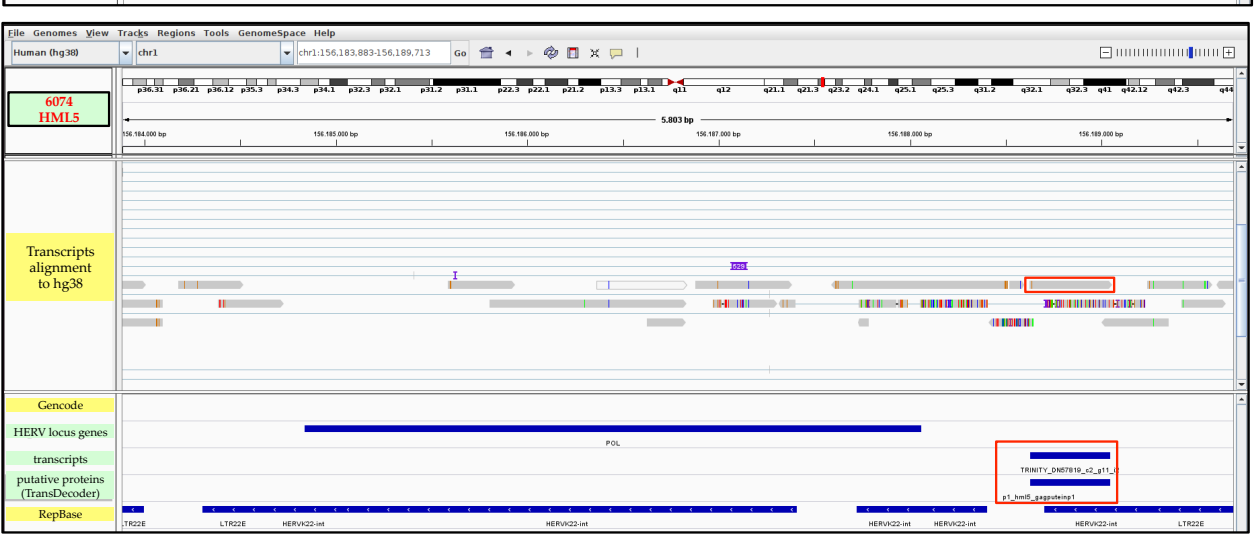

Supplement: Supplementary file 1 [file viruses-12-00481-s001.zip › updated_suppl_files/Figure_S3.pdf]

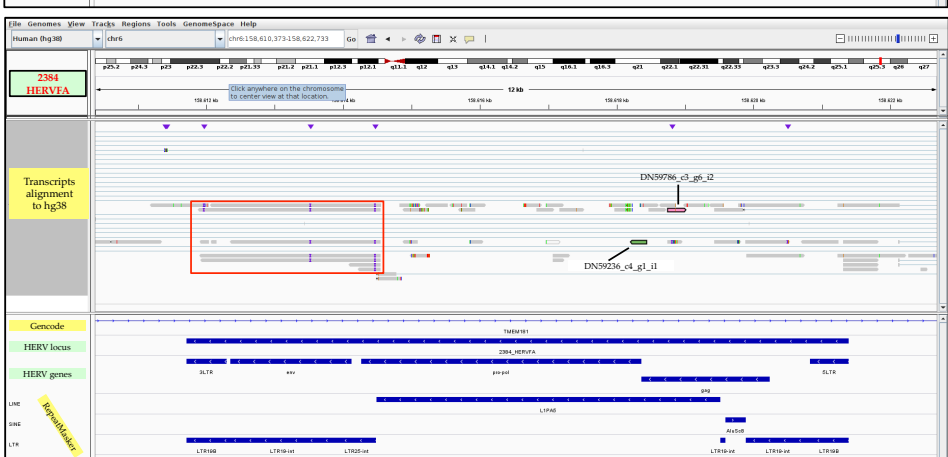

Supplement: Supplementary file 1 [file viruses-12-00481-s001.zip › updated_suppl_files/Figure_S2.pdf]
