# Supplementary material for: Comprehensive Analysis of HERV Transcriptome in HIV+ Cells: Absence of HML2 Activation and General Downregulation of Individual HERV Loci
Source: Viruses. 2020 Apr 23;12(4):481. doi: 10.3390/v12040481 (PMC7232394; doi:10.3390/v12040481)
Supplement: Supplementary file 1 [file Figure_S1.pdf › updated_suppl_files/Figure_S1.pdf]

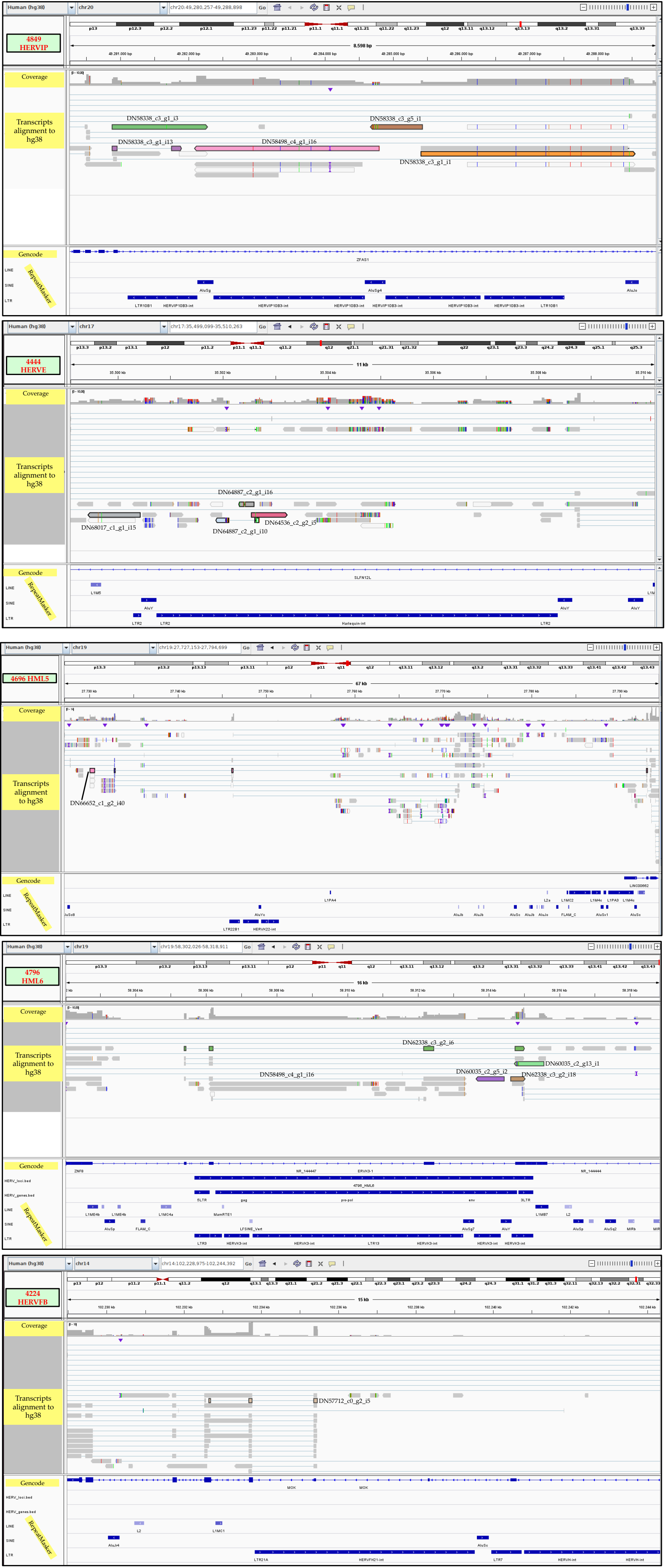

Human (hg38)
chr20
chr20:49,280,257-49,288,898
4849 HERVIP
8,598 bp
Coverage
Transcripts alignment to hg38
DN58338_c3_g1_i3
DN58338_c3_g5_i1
DN58338_c3_g1_i13
DN58498_c4_g1_i16
DN58338_c3_g1_i1
Gencode
RepeatMasker
LINE
SINE
LTR
ZFAS1
AluSg
AluSg4
AluJo
LTR10B1
HERVIP10B3-int
chr17
chr17:35,499,099-35,510,263
4444 HERVE
11 kb
DN64887_c2_g1_i16
DN68017_c1_g1_i15
DN64536_c2_g2_i5
DN64887_c2_g1_i10
SLFN12L
L1M5
AluY
LTR2
Harlequin-int
chr19
chr19:27,727,153-27,794,699
4696 HML5
67 kb
Transcripts alignment to hg38
DN66652_c1_g2_i40
LINC00662
L1PA4
AluYc
LTR22B1
HERVK22-int
chr19:58,302,026-58,318,911
4796 HML6
16 kb
DN62338_c3_g2_i6
DN60035_c2_g13_i1
DN58498_c4_g1_i16
DN60035_c2_g5_i2
DN62338_c3_g1_i18
HERV_loci.bed
HERV_genes.bed
ZNF8
NR_144447
ERVK3-1
4796_HML6
5LTR
gag
pro-pol
env
3LTR
LTR3
HERVK3-int
LTR13
chr14
chr14:102,228,975-102,244,392
4224 HERVFB
15 kb
DN57712_c0_g2_i5
MOK
L2
L1MC1
AluJr4
AluSc
LTR21A
HERVFH21-int
LTR7
HERVH-int
